# Correction: High Rates of O'Nyong Nyong and Chikungunya Virus Transmission in Coastal Kenya

**DOI:** 10.1371/journal.pntd.0003674

**Published:** 2015-04-07

**Authors:** The PLOS Neglected Tropical Diseases Staff

## Notice of Republication

This article was republished on March 2, 2015 to correct an error in Figure 4. The bottom panel was previously omitted. Please download this article again to view the correct version. The originally published, uncorrected article and the republished, corrected article are provided here for reference.

## Supporting Information

S1 File Originally published, uncorrected article. (PDF)

S2 File Republished corrected article. (PDF)
