# Supplementary material for: High Rates of O’Nyong Nyong and Chikungunya Virus Transmission in Coastal Kenya
Source: PLoS Negl Trop Dis. 2015 Feb 6;9(2):e0003436. doi: 10.1371/journal.pntd.0003436 (PMC4319898; doi:10.1371/journal.pntd.0003436)
Supplement: S1 Fig — (DOC) [file pntd.0003436.s001.doc]

**Village No :**  **Household No:**  **Person ID: Study ID:**

**KWALE,MSAMBWENI and KINANGO DISTRICT STUDY: DEMOGRAPHIC QUESTIONNAIRE**

Interviewer Name: __________________________________ Interview Date (DD/MM/YYYY): / /

**Identification of informant- head of household or head spouse**

| A1 | **What is your first name?** |  | | | |
| --- | --- | --- | --- | --- | --- |
| A2 | **What is your second name?** |  | | | |
| A3 | **What is your family name?** |  | | | |
| A4 | **What is your sex?** | □ Male | □ Female | |  |
| A5 | **What month and year were you born?** | / |  | |  |
| A6 | **What was your first language?** | □ Swahili  □ Duruma  □ Digo | □ Kamba  □ Other: __________________  □ Refused | |  |
| A7 | **What is your tribe?** | □ Digo  □ Duruma  □ Kamba |  | |  |
|  |  |  | □ Other: _____________  □ Refused | | |
| A8 | **What is your religion?** | □ Islam/Muslim | □ Other: ______________________________ | | |
|  |  | □ Christianity/Christian | | □ Refused | |

| A9 | **Are you ( informant) married ?** | □ Yes | □ No | □ Refused |
| --- | --- | --- | --- | --- |
| A10 | **Do you have any children?** | □ Yes | □ No | □ Refused |
| A11 | **Do you own or rent the house where you live?** | □ Own | □ Rent | □ Refused |
|  |  | □ Other: ______________________________ | |  |
| A12 | **How many years have you lived in Kwale/Msambweni/Kinango district?** | Years: __ __ | □ Entire Lifetime  □ Don’t Know | □ Refused |
| A13 | **How many years have you lived in your current residence?** | Years: __ __ | □ Entire Lifetime  □ Don’t Know | □ Refused |
| A14 | **How many rooms are there in your house? ( exclude toilet and include outside rooms like kitchen)** | __ __ Rooms |  |  |
| A15 | **How many bedrooms are there in your house?** | __ __ Rooms |  |  |
| A16 | **How many people sleep in the same sleeping room?**  **( highest value per room)** | ____ members |  |  |
| A17 | **How many windows are there in your house?** | __ __ windows |  |  |
| A18 | **Do you sleep close to a window?** | □ Yes | □ No | □ Refused |
| A19 | **Do you sleep under a bed net at night?** | □ Yes | □ No | □ Refused |
| A20 | **Do your children sleep under a bed net at night?** | □ All □ Some | □ None | □ Refused |
| A21 | **Has the bed net been treated ?** | □ Yes | □ No | □ Refused |
| A22 | **What is the type of flooring in your dwelling?**  **( Interviewer should confirm by inspection)** | □ Dirt/earth  □ Wood/plank  □ Tile | □ Other ________ |  |
| A23 | **What is the roof made out of?**  **( Interviewer confirm by inspection)** | □ Natural material  □ Corrugated iron  □ RoofingTiles | □ Other ________ |  |

**A 24 HOUSEHOLD MEMBERS**

| Name 1 | Name 2 | Family Name | Relationship to HoH | Study ID | Date of birth | Sex | Name of school attended | Live here? | Slept here last night? |
| --- | --- | --- | --- | --- | --- | --- | --- | --- | --- |
|  |  |  |  |  |  |  |  |  |  |
|  |  |  |  |  |  |  |  |  |  |
|  |  |  |  |  |  |  |  |  |  |
|  |  |  |  |  |  |  |  |  |  |
|  |  |  |  |  |  |  |  |  |  |
|  |  |  |  |  |  |  |  |  |  |
|  |  |  |  |  |  |  |  |  |  |
|  |  |  |  |  |  |  |  |  |  |
|  |  |  |  |  |  |  |  |  |  |
|  |  |  |  |  |  |  |  |  |  |
|  |  |  |  |  |  |  |  |  |  |
|  |  |  |  |  |  |  |  |  |  |
|  |  |  |  |  |  |  |  |  |  |
|  |  |  |  |  |  |  |  |  |  |
|  |  |  |  |  |  |  |  |  |  |
|  |  |  |  |  |  |  |  |  |  |
|  |  |  |  |  |  |  |  |  |  |
|  |  |  |  |  |  |  |  |  |  |
|  |  |  |  |  |  |  |  |  |  |
|  |  |  |  |  |  |  |  |  |  |
|  |  |  |  |  |  |  |  |  |  |
|  |  |  |  |  |  |  |  |  |  |

**HABITS**

| A25 | **What is your main source of cooking fuel?( Check all that apply)** | □ Electricity  □ Paraffin  □ Gas | □ Firewood  □ Charcoal | □ Solar  □ Refused |
| --- | --- | --- | --- | --- |
|  |  |  | □ Other: ______________________________ | |
| A26 | **What is the principal household source of drinking water?**  **(Choose one)** | □ Piped water in house  □ Piped water in public tap  □ Public Well | □ Rain  □ River/canal | □ Other__________ |
| A27 | **What is your main source of lighting at night?**  **(Choose one)** | □ Electricity line  □ Pressure Lamp  □ Lantern  □ Tin Lamp | □ Fuel Wood  □ Solar electrical battery  □ Candles | □ Kerosene  □ Refused |
|  |  |  | □ Other: ______________________________ | |
| A28 | **Do you own land?** | □ Yes | □ No | □ Refused |
| A29 | **If you answered yes to A28, does anybody in the household work the land?** | □ Yes | □ No | □ Refused |
| A30 | **Do you own a telephone?** | □ Yes | □ No | □ Refused |
| A31 | **Do you own a radio?** | □ Yes | □ No | □ Refused |
| A32 | **Do you own a television?** | □ Yes | □ No | □ Refused |
| A33 | **Do you own a bicycle?** | □ Yes | □ No | □ Refused |
| A34 | **Do you own a motorized vehicle** (e.g. automobile, scooter)**?** | □ Yes | □ No | □ Refused |
| A35 | **Do you have a domestic worker?** | □ Yes | □ No | □ Refused |

| A36 | **Do you have a flush toilet?** | □ Yes | | □ No | | □ Refused | |
| --- | --- | --- | --- | --- | --- | --- | --- |
| A37 | **Do you share a flush toilet?** | □ Yes | | □ No | | □ Refused | |
| A38 | **Do you use a latrine?** | □ Yes | | □ No | | □ Refused | |
| A39 | **Where is your toilet/latrine located?** | □ Inside your house  □ Outside (without  water) | | □ Outside (with water)  □ No Toilet | | □ Refused | |
|  |  |  |  | □ Other: ______________________________ | | | |
| A40 | **How far is your toilet/latrine from your house?** | □ Less than 10 m  from house | | □ 10 m or more from  the house | | □ Refused | |
| A41 | **What was the highest level of schooling that you completed?** | □ Primary □Secondary | □ College  □ University | | □ None | | □ Refused |

**Reporter’s initials: __________**

**Reporter’s signature:__________________________________**
