# Supplementary material for: Correction: High Rates of O'Nyong Nyong and Chikungunya Virus Transmission in Coastal Kenya
Source: PLoS Negl Trop Dis. 2015 Apr 7;9(4):e0003674. doi: 10.1371/journal.pntd.0003674 (PMC4388627; doi:10.1371/journal.pntd.0003674)
Supplement: S2 File — (PDF) [file pntd.0003674.s002.pdf]

PLOS | NEGLECTED TROPICAL DISEASES

RESEARCH ARTICLE

High Rates of O'Nyong Nyong and Chikungunya Virus Transmission in Coastal Kenya

A. Desiree LaBeaud[1,2¤]*, Tamara Banda[1], Julie Brichard[1], Eric M. Muchiri[3], Peter L. Mungai[2], Francis M. Mutuku[4], Erin Borland[5], Ginny Gildengorin[1], Sarah Pfeil[1], Crystal Y. Teng[1], Kristin Long[6], Mark Heise[6], Ann M. Powers[5], Uriel Kitron[7], Charles H. King[2]

1 Children's Hospital Oakland Research Institute, Oakland, California, United States of America, **2** Case Western Reserve University, Cleveland, Ohio, United States of America, **3** Division of Vector Borne and Neglected Tropical Diseases, Ministry of Health, Msambweni, Kenya, **4** Technical University of Mombasa, Mombasa, Kenya, **5** CDC, Fort Collins, Colorado, United States of America, **6** University of NC, Chapel Hill, North Carolina, United States of America, **7** Emory University, Atlanta, Georgia, United Statas of America

¤ Current address: Department of Pediatrics, Division of infectious diseases, Stanford University, Stanford, California, United States of America
* dlabeaud@stanford.edu

OPEN ACCESS

Citation: LaBeaud AD, Banda T, Brichard J, Muchiri EM, Mungai PL, Mutuku FM, et al. (2015) High Rates of O'Nyong Nyong and Chikungunya Virus Transmission in Coastal Kenya. PLoS Negl Trop Dis 9(2): e0003436. doi:10.1371/journal.pntd.0003436

Editor: Matthew Kasper, U.S. Naval Medical Research Unit Six, UNITED STATES

Received: July 19, 2014

Accepted: November 23, 2014

Published: February 6, 2015

Copyright: This is an open access article, free of all copyright, and may be freely reproduced, distributed, transmitted, modified, built upon, or otherwise used by anyone for any lawful purpose. The work is made available under the Creative Commons CC0 public domain dedication.

Data Availability Statement: We ask very detailed information about age, sex, occupation and take the X and Y coordinates of the household by GPS, so in essence someone with that data could unmask all of our participants. We have provided data that does not contain any PHI in S1 Data set, but our entire dataset cannot be provided for privacy and security reasons. The U.S. IRB contact is: Brigid Roy, CHRCO IRB, irb@mail.cho.org, 1-5010-428-3173. The KEMRI IRB contact is Dr. Elizabeth Anne Bukusi, KEMRI, ebukusi@kemri.org, +254202712537.

Funding: This work was supported by PNWRCE AI81680:RP012 (PI: LaBeaud), NIH AI102918

Abstract

Background

Chikungunya virus (CHIKV) and o'nyong nyong virus (ONNV) are mosquito-borne alphaviruses endemic in East Africa that cause acute febrile illness and arthritis. The objectives of this study were to measure the seroprevalence of CHIKV and ONNV in coastal Kenya and link it to demographics and other risk factors.

Methodology

Demographic and exposure questionnaires were administered to 1,848 participants recruited from two village clusters (Milalani-Nganja and Vuga) in 2009. Sera were tested for alphavirus exposure using standardized CHIKV IgG ELISA protocols and confirmed with plaque reduction neutralization tests (PRNT). Logistic regression models were used to determine the variables associated with seropositivity. Weighted K test for global clustering of houses with alphavirus positive participants was performed for distance ranges of 50–1,000 meters, and G* statistic and kernel density mapping were used to identify locations of higher seroprevalence.

Principal Findings

486 (26%) participants were seropositive by IgG ELISA. Of 443 PRNT confirmed positives, 25 samples (6%) were CHIKV+, 250 samples (56%) were ONNV+, and 168 samples (38%) had high titers for both. Age was significantly associated with seropositivity (OR 1.01 per year, 95% C.I. 1.00–1.01); however, younger adults were more likely to be seropositive than older adults. Males were less likely to be seropositive ($p<0.05$; OR 0.79, 95% C.I.

(PI: LaBeaud), and NIH/NSF EEID TW008067 (PIs: Kitron and King). The funders had no role in study design, data collection and analysis, decision to publish, or preparation of the manuscript.

Competing Interests: The authors have declared that no competing interests exist.

0.64–0.97). Adults who owned a bicycle ($p<0.05$; OR 1.37, 95% C.I. 1.00–1.85) or motor vehicle ($p<0.05$; OR 4.64, 95% C.I. 1.19–18.05) were more likely to be seropositive. Spatial analysis demonstrated hotspots of transmission within each village and clustering among local households in Milalani-Nganja, peaking at the 200—00m range.

Conclusions/Significance

Alphavirus exposure, particularly ONNV exposure, is common in coastal Kenya with ongoing interepidemic transmission of both ONNV and CHIKV. Women and adults were more likely to be seropositive. Household location may be a defining factor for the ecology of alphaviral transmission in this region.

Author Summary

Alphaviruses, such as chikungunya and o'nyong nyong viruses, are likely important causes of human disease in endemic regions, but are often misdiagnosed as malaria in the acute care setting. Our objective was to uncover the burden of alphavirus exposure in our study region, rural coastal Kenya. Of 1848 participants tested, 26% were seropositive by screening ELISA, demonstrating intense transmission to humans in this area. Surprisingly, confirmatory PRNT testing revealed that the majority of alphavirus exposures were due to o'nyong nyong virus, rather than chikungunya virus. Both CHIKV and ONNV antibodies were confirmed in young children, demonstrating undocumented and ongoing transmission in this region. Of the examined risk factors, older age and female gender were associated with alphavirus seropositivity.

Introduction

Alphaviruses are endemic to many regions of Kenya; however, due to limited surveillance and documentation during and in between known outbreaks, the alphaviral burden is yet to be fully recognized. O'nyong-nyong virus (ONNV) and chikungunya virus (CHIKV) are closely related alphaviruses in the Semliki Forest antigenic complex [1]. CHIKV was initially isolated from a febrile person in Tanzania in 1953 [2] and small outbreaks occurred in Kenya in the late 1900s. In 2004, CHIKV re-emerged in Kenya and subsequently spread eastward around the Indian Ocean countries, causing a severe epidemic, and resulting in significant morbidity that heavily taxed the healthcare and public health infrastructure in many regions [3]. More recently in 2013, CHIKV spread to the Americas, where it continues to cause outbreaks in many Caribbean islands with over 100,000 cases reported within the first 6 months [4]. ONNV was initially isolated in Northern Uganda from anopheline mosquitoes and human serum during a 1959 epidemic [5]. ONNV has been associated with relatively few but large-scale epidemics. In 1996, ONNV resurfaced in Southern Uganda, causing major isolated epidemics, but was last reported in Kenya in 1961 [6].

Both CHIKV and ONNV cause febrile illness in humans. Clinically, the symptoms of CHIKV are difficult to distinguish from those of dengue fever [7, 8]. Because malaria, dengue and CHIKV co-circulate in many regions, CHIKV is often misdiagnosed and under recognized [9–11]. CHIKV infections are primarily characterized by fever and polyarthralgia, favoring the small joints and sites of previous injuries, but may also be associated with headache, nausea,

vomiting, myalgia, lymphadenopathy, and rash [12]. The clinical features of ONNV infections include a low-grade fever, symmetrical polyarthralgia, lymphadenopathy, generalized papular or maculopapular exanthema, and joint pain [6]. Fever from ONNV may abate and recrudesce after a few days, giving rise to a "saddleback" fever curve [6, 7, 12]; this is less common with CHIKV. Symptoms may last from 1 week to several months and can result in significant morbidity [13].

While there is growing research interest in CHIKV as it spreads within Europe and into the Americas, the role of ONNV in endemic regions, especially in sub-Saharan Africa, remains unclear despite its close relationship to CHIKV [1]. A small number of serosurveys have provided the limited available information about the relationships of CHIK and ONN viruses [14–16]. Prior serostudies in Kenya have demonstrated a large potential burden of alphaviruses, but few studies have taken place since the large CHIKV outbreak of 2004 to determine whether interepidemic transmission is ongoing in Kenya [17]. In this study we aimed to quantify the prevalence of human exposure to alphaviruses in the coast of Kenya and determine whether CHIKV and/or ONNV were circulating.

Materials and Methods

Ethics statement

All participants in this study consented to participation in their own language. Written consent was obtained from all adult participants; children provided assent with parental written consent. For the parent study of community infectious burden, IRB protocols were approved by Case Western Reserve University (protocol # 11–07–42) and the Kenya Medical Research Institute (SSC 2611). In addition, for this study, IRB approval was obtained from Children's Hospital & Research Center Oakland (CHRCO).

Study population

This study was performed using banked serum specimens from an NIH-funded study on polyparasitism in coastal Kenya (Eco-epidemiology of schistosomiasis, malaria and polyparasitism in coastal Kenya; PIs: Kitron and King), as described by Bustinduy and Bisanzio [18, 19]. In preparation for this study, a census of the local community was undertaken, during which information on household, socioeconomic status, and demographics was collected (S1 Fig.). All community households and all household residents over the age of 1 year were eligible for participation. The location of each household was geo-referenced by GPS for later spatial analysis. Sera were collected, stored and later shipped to CHRCO on dry ice for serologic testing of study subjects' alphavirus exposure. Two village clusters were studied for the current analysis; Milalani-Nganja (4.473036°S and 39.459060°E) and Vuga (4.186887°S and 39.507513°E) located in Kwale county, coastal Kenya.

Test antigen produced from 181/25 strain Chikungunya virus

Vero81 cells were infected with CHIKV strain 181/25 at an MOI = 0.01 in a 30 ml volume in T175 flasks and the infection was allowed to progress 48 hours until the monolayer was destroyed. The supernatant was harvested from flasks and centrifuged at 3000 rpm at 4°C for 20 minutes. A 40 ml aliquot of the infected cell supernatant was added to one of four new T175 tissue culture flasks, followed by addition of 1.3 ml binary ethylenimine (BEI) to a final concentration of 3 mM to inactivate the virus [20]. The flasks were placed in a rotator at 90 rpm at 37°C for 24 hours. The supernatant was removed to an ultra-centrifuge tube, underlayed with 5.0 ml 20% sucrose, then centrifuged for 3 hours at 24,000 rpm at 4°C. Supernatant was decanted

completely and 500 ul of sterile PBS was added to each tube. Next, 17 ul of BEI was added to the tubes and tubes were covered with parafilm. Inactivation was performed on a rotator at 90 rpm at 37°C for 24 hours. A 25 ul aliquot of cold stop solution was finally added and the mixture was allowed to sit at 4°C for 30 minutes. P1000 tips were used to scrape the virus off of bottom of tube into PBS, which was then mixed by pipetting thoroughly. The virus obtained from all tubes was combined into a single conical tube at 9.0 ml total volume with PBS.

Enzyme-linked immunosorbent assay (ELISA) testing

Sera were separated from whole blood specimens and screened for anti-alphaviral IgG antibodies using a direct enzyme-linked immunosorbent assay (ELISA) with CHIKV 181/25 antigen. Briefly, 96-well polystyrene ELISA plates were coated with 1ug/ml of vaccine strain 181/ 25 CHIKV antigen (see above for production), and were held overnight at 4°C. After four washes with phosphate-buffered saline (PBS) with 0.05% Tween-20 (PBS-T), non-specific binding was blocked by adding 5% non-fat dry milk in PBS with 0.5% Tween-20 and plates were incubated for 60 minutes at 37°C. Sample sera were added at a 1:100 dilution and plates were incubated for 60 minutes at 37°C. Each serum sample was run in duplicate. A 1:1,000 dilution of conjugated goat anti-human IgG (109–055–098 AP-AffiniPure Goat Anti-Human IgG, Fcγ Frag Specific, Jackson Immunoresearch Laboratories, Inc., West Grove, Pa.) was used as the second antibody. Plates were incubated for 60 minutes at 37°C. Presence of CHIKV antibodies was detected by adding alkaline phosphatase substrate (Sigma Aldrich), and the absorbance was read at 405 nm. Positive samples required a mean optical density (OD) value $\geq$ 0.2 above that of the negative control for each ELISA plate. The range of OD values for positive serum specimens in this study was 0.3–1.5 above that of the negative control. Positive controls were obtained by creating a pool of sera that had tested positive by ELISA and concurrently confirmed positive by plaque reduction neutralization testing (PRNT), as detailed below. Negative controls were obtained by creating a pool of sera that had tested negative for alphavirus exposure by ELISA and PRNT.

Plaque reduction neutralization testing

A subset of samples (all ELISA positives and equivocals, and a small random set of ELISA negatives) was subjected to PRNT confirmatory testing to verify ELISA screening results and determine whether seropositivity was due to CHIKV or ONNV infection. Briefly, the samples were heat-inactivated for 30 min at 56°C. Using 96 well cell culture cluster plates (Corning, Corning, NY) samples were serially diluted two-fold in Dulbecco's Minimum Essential Medium supplemented with 10% FBS, 100 U/ml of penicillin, and 100 mg/ml of streptomycin (Gibco, Carlsbad, CA) for a final volume of 60 ul per well. A suspension of 50 PFU/60 uL (CHIKV COM125 strain) was then mixed 1:1 with the diluted serum samples and the mixture incubated for 1 h at 37°C. The serum-virus suspension was then transferred onto 12-well cell culture plates (Corning, Corning, NY) containing a confluent monolayer of Vero cells. The virus/cell mixture was incubated for 1 h at 37°C with the plates agitated every 15 min. Each well was then overlaid with a 0.4% Genepure LE agarose/DMEM medium layer (ISC BioExpress, Kaysville, UT) and plates were incubated at 37°C for 48h for CHIKV and 72h for ONNV. The agarose layer was removed and the wells were covered with a fixative/staining solution (40% methanol, 0.25% crystal violet) for 10 min. Plates were then rinsed with water. Plaques were counted and titers were calculated and expressed as the reciprocal of serum dilution yielding a >80% reduction ($PRNT_{80}$) in the number of plaques. Prevalence results reported in this analysis are based on PRNT results. Samples were considered CHIKV PRNT positive if the titer was >20 and the ONNV titer was < four-fold lower than the CHIKV titer. Because there is a unique one-way

antigenic cross-reactivity between CHIKV and ONNV [21], a sample was designated ONNV positive if its titer was >20 and two-fold or greater than the CHIKV titer [22].

Malaria screening

Fingerprick specimens for all subjects were tested for *Plasmodium falciparum* malaria using a commercial rapid circulating antigen test (ICT Diagnostics, Australia).

Data analysis

Descriptive analyses on the demographic and household variables were conducted on the sample across the villages and for different age groups. Children were defined as those being less than 16 years of age, while adults were those individuals that were 16 years of age or older. Analyses included means, measures of variability, proportions and confidence intervals. Human alphavirus seropositivity was the principal outcome measure for the study. Initial exploratory assessment of differences in categorical measures with alphaviral seropositivity was done using chi–square or Fisher's exact test. We used Student's t-test for comparisons of continuous measures. Logistic regression models were used as the primary analyses with the binary outcome of alphavirus infection (seropositive vs seronegative). These models adjusted for the differences between villages (Vuga, Milalani-Nganja). A significance level of 0.05 was used for all statistical tests. Subset analysis was performed for the children (under 16 years) and adults. The statistical software package SAS v 9.3 (SAS Institute, Inc, Cary NC) was used to analyze the data. The unweighted and weighted K test (for global clustering of houses and then superclustering of seropositivity, respectively) and kernel density mapping were performed for distance ranges of 50–1,000 meters using ArcMap 10.1 (ESRI, Redlands, CA). Getis G^* statistic testing, with correction for multiple comparisons, was performed using ClusterSeer (BioMedware, Ann Arbor, MI).

Results

Of the 1864 samples initially tested for anti-alphavirus IgG antibodies by ELISA, 413 (22%) were positive, 134 (7%) were equivocal and 1317 (70%) were negative. Ninety percent (371/413) of ELISA positives, eighty seven percent (117/134) of ELISA equivocal and 4.5 percent (59/1317) of ELISA negatives were PRNT tested. In the 83 cases in which the PRNT result differed from the ELISA result, the PRNT result was used as the final result and the sixteen equivocal samples that were not PRNT tested were removed from the final analysis. Eighty four percent (313/371) of ELISA positive, 89% (105/117) of ELISA equivocal and 42% (25/59) of ELISA negative were confirmed positive by PRNT. In our final analysis, 486 out of 1848 (26%; 95% CI 24.3%–28.4%) participants were positive for anti-alphavirus IgG antibodies by ELISA and 443 were confirmed positive by PRNT (Fig. 1). PRNT data for all of the ELISA positive and equivocal samples are not available because of specimen volume limitations inhibiting both ONNV and CHIKV PRNT to be performed.

Two hundred seventy (56%) alphavirus-exposed participants were from Milalani-Nganja. Using analysis by location, village of residence was not significantly associated with seropositivity (P = 0.458). 25.4% (95% CI 22.5%–28.5%) of Vuga participants were seropositive vs. 27.0% (95% CI 24.3–29.9%) in Milalani-Nganja. Three hundred fifty-three (72%) of seropositives were adults. Age was significantly associated with seropositivity (OR 3.08 adults vs. children, 95% CI 2.42–3.94; OR1.01 per year controlling for village and gender, 95% C.I 1.00–1.01). Mean age of alphavirus-exposed subjects was 27 years (range 1–79 y). Males were less likely to be seropositive (p = 0.025; OR 0.79, 95% C.I 0.064–0.97). Adults who owned a bicycle (p = 0.046; OR 1.37, 95% C.I 1.00–1.85) or motor vehicle (p = 0.027; OR 4.64, 95% C.I 1.19–18.05) were more likely to be seropositive (Table 1). In multivariate analysis older age and

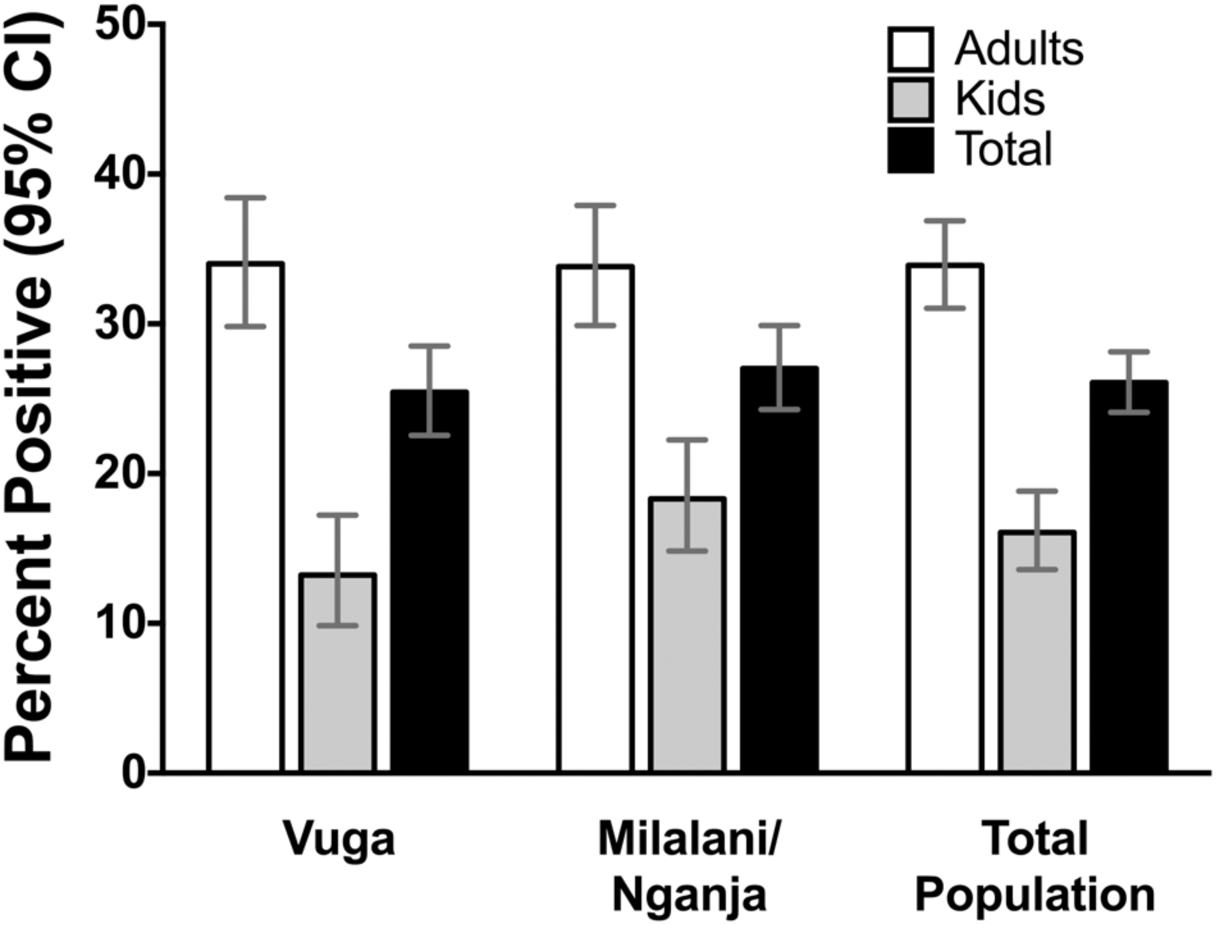

Figure 1. Alphavirus seroprevalence as measured by ELISA displayed by village and age group. 95% CI indicated by bars.

doi:10.1371/journal.pntd.0003436.g001

female gender were associated with seropositivity (Table 2). The majority of seropositives (358, 74%) reported bednet use. As is typical for this region, the majority of seropositives had earthen flooring, thatched roofs, and used firewood as cooking fuel (65%, 77%, and 94%, respectively).

In children (<16 years, N = 711), the only variable significantly associated with seropositivity was age (p<0.001), with older children being more likely to be seropositive. Among adults (N = 1139), female gender was associated with seropositivity (p = 0.018; OR 1.38, 95% C.I. 1.05–1.78). Age was also associated with seropositivity within the adult subgroup, however in this case it was younger adults who were more likely to be seropositive than older adults (p<0.001, OR 0.98 95% CI.97–0.99)(Fig. 2).

Of 443 PRNT confirmed positives, 250 samples (56% of positives, 13% of total) were positive for ONNV, 25 samples (6% of positives, 1% of total) were positive for CHIKV, and 168 samples (38% of positives, 9% of total) had high PRNT titers for both CHIKV and ONNV. A positive CHIKV test was more likely to be from Vuga (Vuga 76% vs. Milalani-Nganja 24%), whereas a positive ONNV test was more likely to be from Mililani-Nganja (Milalani-Nganja 58% vs. Vuga 42%), though this was not statistically significant. If a more conservative 4-fold titer cutoff was used instead of 2-fold cutoff to designate ONNV antibody positivity, 37% and 57% would be considered ONNV and undetermined alphavirus antibody positive, respectively. PRNT titer to CHIKV or ONNV did not drop over time until after age 70 years (Fig. 3).

Table 1. Bivariate analysis of factors associated with alphaviral seropositivity controlling for village of residence.

Variable	Odds Ratio	95% Confidence Limits	P Value
**Total Population**			
Age (adults vs. children)	3.08	2.42–3.94	<0.001
Sex (female vs. male)	1.27	1.03–1.57	0.025
Own Bicycle	1.29	1.01–1.65	0.038
Number of sleepers in household	0.89	0.81–0.97	0.010
**Children Only**			
Number of sleepers in household	0.79	0.66–0.95	0.011
Age	1.49	1.37–1.64	<0.001
**Adults Only**			
Sex (female vs. male)	1.38	1.06–1.80	0.018
Bicycle	1.37	1.01–1.85	0.046
Own car	4.64	1.19–18.05	0.027
Age	0.98	0.97–0.99	<0.001

doi:10.1371/journal.pntd.0003436.t001

For those who were ONNV antibody positive (N = 250), mean age was 28.4 years (range 2–75y) and the majority (75%) were adults. Fifty-eight percent of ONNV-exposed participants came from Milalani-Nganja, and 64% were female. For those who were CHIKV antibody positive (N = 25), mean age was 21.7 years (range 7–54y) and the majority (75%) were adults. Only 24% of CHIKV-exposed participants came from Milalani-Nganja and 70% were female. The mean age of the undetermined group was 27.9 years (range 1–79y). We did not see a significant association between current malaria and ONNV exposure in our study subjects despite being likely transmitted by the same *Anopheles* spp. mosquitoes.

Spatial analysis

Geographic Information Systems (GIS)-based spatial analysis (Figs. 4 and 5) was performed for seropositivity in the separate village locations, Milalani-Nganja (contiguous villages) and Vuga. Following an assessment of the baseline clustering of community households (using the unweighted K-statistic) the weighted K test for global clustering of seroprevalence in alphavirus positive households was performed for distance ranges 50–1000 meters. This analysis showed a significant additional clustering effect for household alphavirus seroprevalence within the combined Milalani-Nganja landscape in the 200–500m range. Figs. 4 and 5 demonstrate the statistical significance of the observed local and global clustering of alphaviral seroprevalence adjusted for aggregation of households across the study landscape. Fig. 4 displays heat maps

Table 2. Multivariate analysis of alphavirus seropositivity and ONNV-only seropositivity.

Variable	Odds Ratio	95% Confidence Limits		P Value
Alphavirus Positive				
Age	1.01	1.00	1.01	0.006
Sex: Female vs. Male	1.25	1.01	1.54	0.044
Village: Milalani/Nganja vs. Vuga	1.09	0.89	1.35	0.407
ONNV Positive				
Age	1.01	1.00	1.02	0.002
Sex: Female vs. Male	1.41	1.06	1.85	0.018
Village: Milalani/Nganja vs. Vuga	1.13	0.86	1.48	0.393

doi:10.1371/journal.pntd.0003436.t002

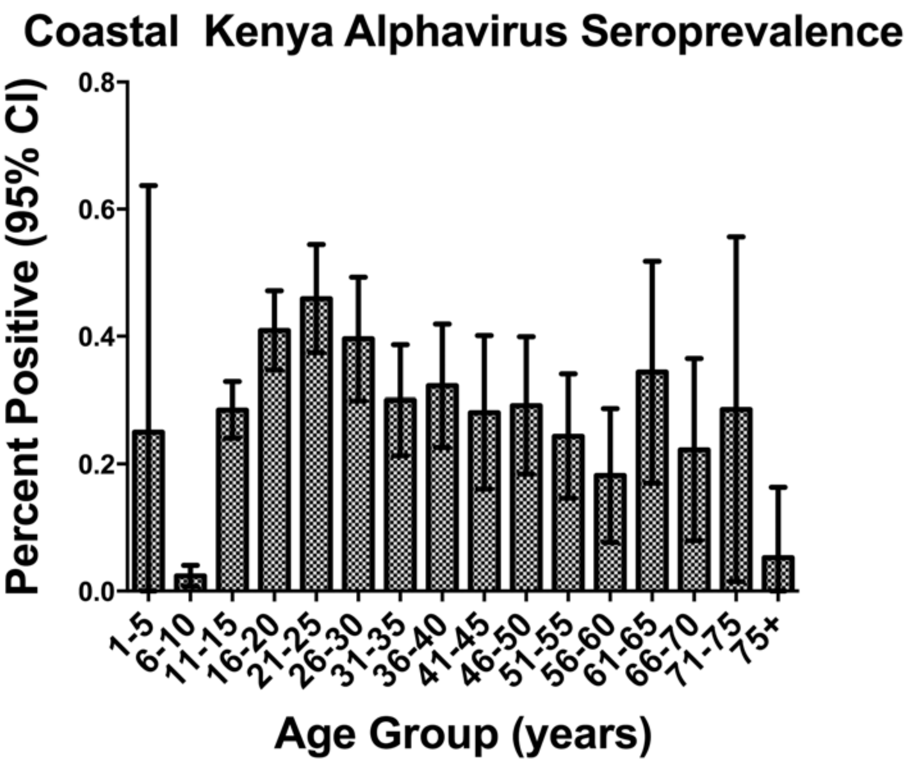

Figure 2. Alphavirus seroprevalence as measured by ELISA displayed by age. 95% CI indicated by bars.

doi:10.1371/journal.pntd.0003436.g002

for both villages and shows regions within both villages with seropositive households. The G^* local statistic indicates a significant hot spot clusters (red circles) of high alphaviral seroprevalence houses in Nganja (scale was 200m)(Fig. 5). Blue circles in Milalani indicate significant relative clustering of negative households (cold spots) in the adjacent area to the southwest. The G^* local statistic indicates a few significant hot spot clusters of high density houses in the south central end of Vuga (scale of 200m)(Fig. 5).

Discussion

Alphavirus exposure, particularly ONNV exposure, was found to be common in coastal Kenya, despite little previous public health attention or research. Evidence of high transmission rates for ONNV was unexpected, given that the last known outbreak in this region was in 1961. The fact that multiple children and young adults were exposed shows that ONNV transmission has occurred in the past five years and remains undetected or poorly reported within the clinical setting. Not surprisingly, children were less likely to be seropositive than adults, likely due to fewer years of mosquito exposure. This suggests that there is endemic circulation with additive risk for exposure over time. Interestingly, younger adults were more likely to be exposed than older adults in this study; however, we did not find evidence that older adults were losing their titers over time (Fig. 3). These two findings suggest an increase in transmission risk of alphaviruses within the last 3 decades in these communities.

Women were more likely to be exposed to alphaviruses in this study. The epidemic and urban vectors for CHIKV are highly anthropophilic and are daytime or crepuscular feeders. It may be that because of cultural roles in this region, women in this community are more likely

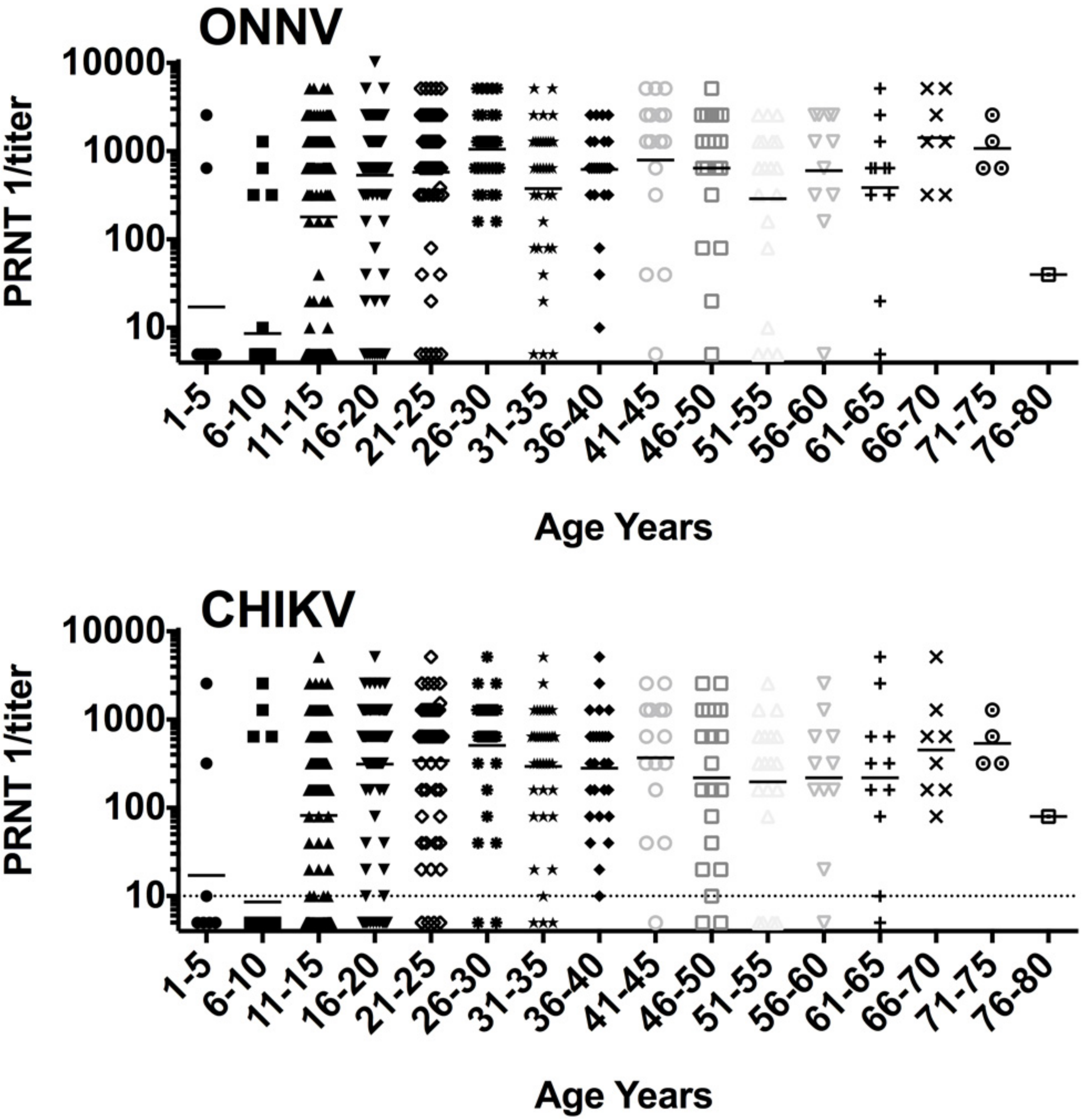

Figure 3. Plaque reduction neutralization titer results by age. ONNV (top) and CHIKV (bottom) titers by 5 year age group. Geometric means indicated by horizontal bars.

doi:10.1371/journal.pntd.0003436.g003

to stay around the homestead where *Aedes* are blood seeking, while men are in the fields, tending to animal herds, or away from home earning livelihoods in more urban areas. There was no significant difference in seroprevalence between those who slept under a bed net and those who did not, even though the majority of alphaviral infections we identified were ONNV which, unlike CHIKV, is spread by night-feeding *Anopheles* spp. Although ONNV is transmitted by the malaria vector, *Anopheles gambiae* [23], we did not find any association between ONNV seropositivity and concurrent malaria infection. The temporal scales used in testing for

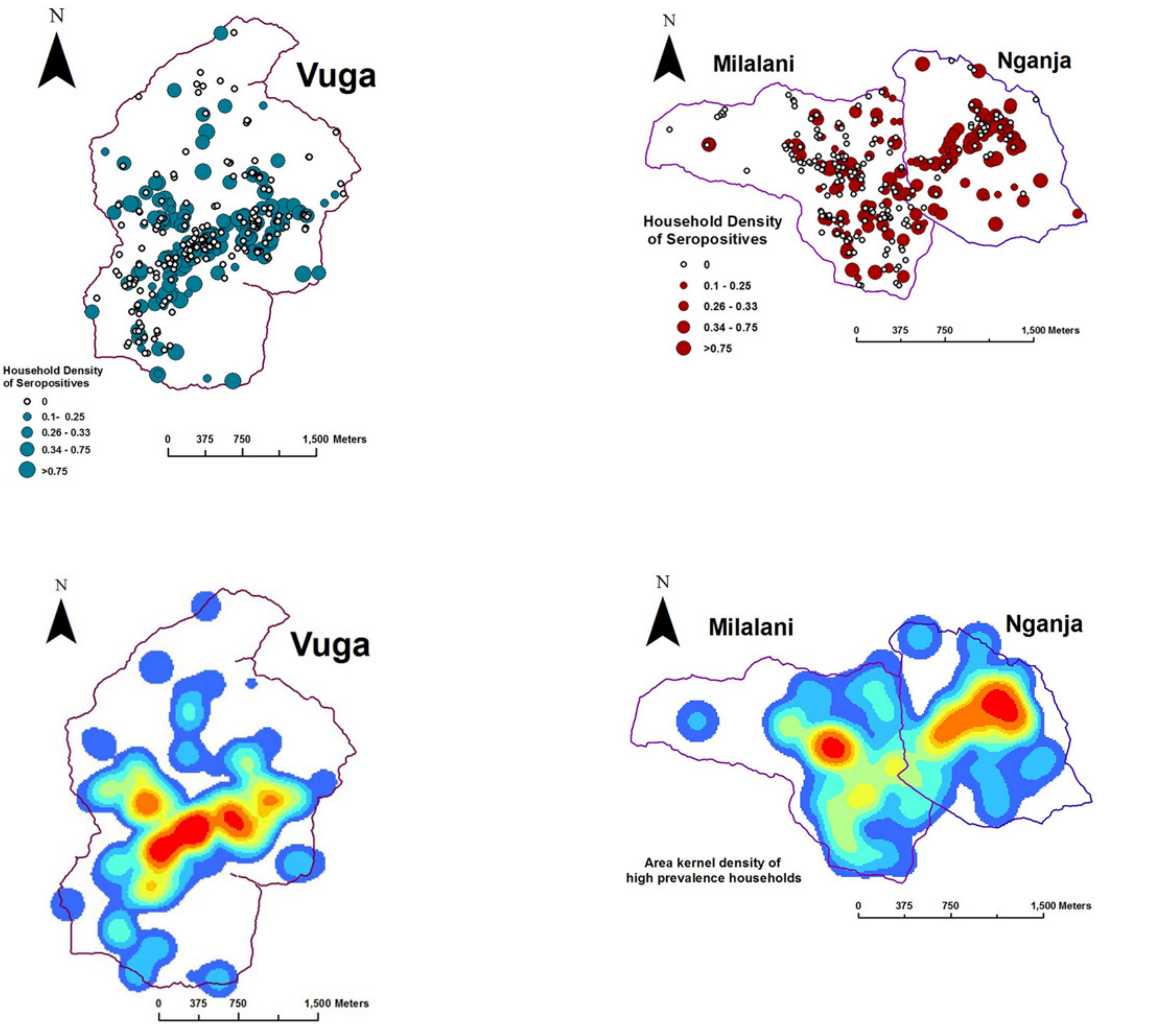

Figure 4. Analytic maps of alphavirus seropositivity in Milalani-Nganja (right) and Vuga (left). The top panels show household locations as dots proportional to the household density of seropositives; the bottom panels show the kernel density estimates for alphaviral seropositivity as a color range, with red having the highest vales and blue the lowest values.

doi:10.1371/journal.pntd.0003436.g004

these two infections were dissimilar—we examined *prior ONNV* exposure via IgG antibody testing, whereas *active P. falciparum* infection was documented by circulating antigen testing and this may explain the lack of association.

Spatial analysis demonstrated global clustering of positive homesteads in the larger Milalani-Nganja complex of villages, but not in the smaller Vuga community. Local clustering analysis further identified 'hotspots' and 'coldspots' of exposure within each village. Where observed, global clustering was most prominent at the 250–400m ranges, further suggesting

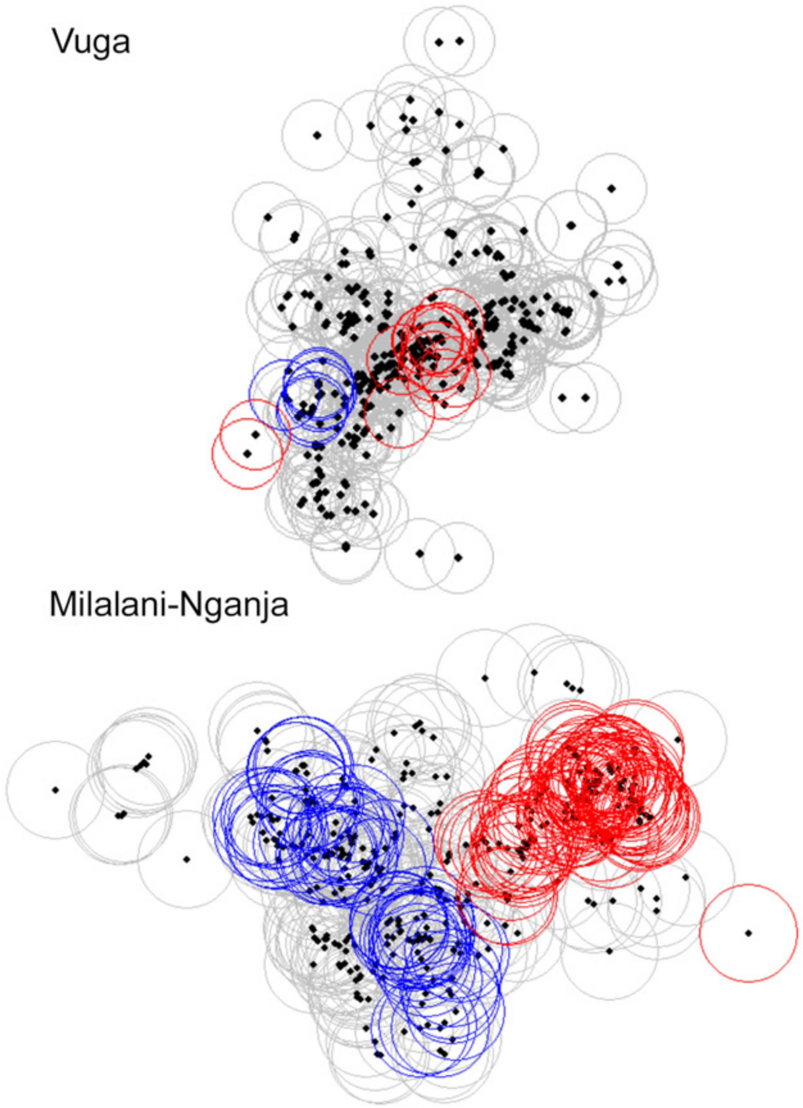

Figure 5. G*-statistic maps of Vuga (top) and Milalani-Nganja (bottom) alphavirus clustering. Clusters of low values are demonstrated in blue circles and clusters of high values are in red circles.

doi:10.1371/journal.pntd.0003436.g005

that there are local factors that influence *Aedes* and *Anopheles* habitats and insect abundance. Although we did not include common daytime gathering points in our local cluster analysis, our spatial analysis suggests that household location is one of the defining factors for CHIKV and ONNV transmission in these villages. Data from Vuga showed local but not global clustering, suggesting that the household factor in Vuga might be less influential than exposure in a shared, more central location. Adults who owned a bicycle or motor vehicle were more likely to be seropositive and this may signal that greater mobility adds a greater level of alphaviral exposure, probably beyond the household/village landscape that was assessed. Individual risk for alphaviral exposure likely includes both household and daytime exposures.

Many of our samples had high titer ($\geq$20) to both ONNV and CHIKV by PRNT. The presence of antibody titers against both viruses could be due to several factors: 1) both viruses could be co-circulating in the region, 2) continued evolution of ONNV has made it

antigenically more similar to CHIKV than previously reported resulting in greater cross neutralization (a one-way cross reactivity between CHIKV and ONNV has previously been documented with antibodies generated against ONNV typically recognizing only ONNV and not CHIKV)(21), 3) another alphavirus that induces antibodies that cross-neutralize both CHIKV and ONNV could be circulating in the area. Individuals who had high titer to both CHIKV and ONNV were relatively young (mean age 28 y), which makes double exposure to both viruses less likely and suggests circulation of another cross-reactive alphavirus or an evolved ONNV lineage. Determination of the exact cause of comparable titers against both viruses requires additional studies with isolation of virus for biological characterization.

Our study has several limitations. We used biobanked sera taken as part of a separate study on parasites; therefore we do not have information on mosquito avoidance or exposures in our participants. We were unable to trap vectors for viral testing in this study and so cannot associate vector type abundance or mosquito infection rates with human data. Due to cost, time, and labor considerations, PRNT could not be performed on the entire study sample set (547 of 1862 samples were tested). As expected, the screening ELISA had several false positives and false negatives detected by PRNT testing, so there may have been misclassification of some participants in our overall survey sample, which would bias our results to underestimation of prevalence: if the 42% false negative rate was applied to our total sample set, our alphaviral prevalence estimates would become 52% (966/1864). Nonspecific false positives from sticky serum can occur, but should have been corrected by PRNT testing. Also some ELISA positive individuals who were PRNT negative may not truly be false positives but may have been exposed but not mounted neutralizing antibodies responses. However, because of the robust size of our sample, we feel that our conclusions are well supported. Finally, a large subset of PRNT positives (38%) could not distinguish between CHIKV and ONNV exposure, which raises the question whether another cross-reacting alphavirus might be co-circulating (although the remaining SF complex viruses are more distantly related genetically).

Acute disease due to CHIKV or ONNV is very infrequently diagnosed in this region of Kenya, as most febrile illness is thought to be due to malaria (9). Given that our data suggests at least 26% (486/1848) of the population is exposed to CHIKV or ONNV, it is likely that a substantial proportion of febrile disease due to alphaviruses is being missed in this clinical setting. CHIKV infected patients have been shown to be more likely to receive inappropriate antibacterial or antimalarial therapy than other febrile patients (10). This has important implications for individual disease management and prevention, and for the emergence of antibacterial or antimalarial drug resistance.

In conclusion, CHIKV and ONNV infections were found to be common in coastal Kenya. It is likely that both CHIKV and ONNV infections are causing human disease that is going undiagnosed in this area. Unrecognized ONNV transmission, in particular, has been ongoing and underappreciated in this region. Among local residents, women and adults were found to be more likely to have been exposed. Finally, household location may be one of the defining factors for the ecology of alphaviral transmission in this region, given our spatial analysis results, and the fact that anophelines transmit ONNV and are associated with biting in homes.

Supporting Information

S1 Fig. Study questionnaire used for data collection.
(DOC)

S1 Data set. De-identified study dataset.
(XLS)

S1 Checklist. STROBE checklist.
(DOC)

Acknowledgments

We would like to thank the individuals who participated in this study and the Kenyan field teams. We would also like to thank David Vu for graphics assistance.

Author Contributions

Conceived and designed the experiments: ADL CHK. Performed the experiments: ADL EB TB SP CT KL EMM PLM FMM. Analyzed the data: ADL TB JB SP CT GG EB AMP CHK. Contributed reagents/materials/analysis tools: KL MH EB AMP. Wrote the paper: ADL TB JB EB GG KL MH AP UK CHK. Field work: ADL PLM FMM EMM UK CHK.

References

1. Powers AM, Brault AC, Tesh RB and Weaver SC. (2000) Re-emergence of Chikungunya and O'nyong-nyong viruses: evidence for distinct geographical lineages and distant evolutionary relationships. J Gen Virol 81:471–479. PMID: 10644846
2. Robinson MC. (1955) An epidemic of virus disease in Southern province, Tanganyika Territory, in 1952–53. Clinical features. Trans R Soc Trop Med Hyg 49: 28–32. doi: 10.1016/0035-9203(55)90080-8 PMID: 14373834
3. Staples JE, Breiman RF, Powers AM. (2009) Chikungunya Fever: An Epidemiological Review of a Re-Emerging Infectious Disease. Clin Infect Dis 49: 942–948. doi: 10.1086/605496 PMID: 19663604
4. Pan American Health Organization (Paho) (2014) Chikungunya, Statistics: Number of reported cases of Chikungunya fever in the Americas. Available: http://www.paho.org/hq/index.php?option = com_content&view = article&id=9053&Itemid=39843
5. Haddow A.J., Davies C.W., Walker A.J.. (1960) O'nyong nyong fever: an epidemic virus disease in West Africa: Introduction. Trans. R. Soc. Trop. Med. Hyg., 54: 517–522. doi: 10.1016/0035-9203(60)90025-0
6. Lanciotti RS, Ludwig ML, Rwaguma EB, Lutwama JJ, Kram TM et al. (1998) Emergence of epidemic O'nyong-nyong fever in Uganda after a 35-year absence: genetic characterization of the virus. Virology 252: 258–268. doi: 10.1006/viro.1998.9437 PMID: 9875334
7. CDC Fact Sheet: Chikungunya. Available: http://www.cdc.gov/chikungunya/symptoms/.
8. CDC Fact Sheet: Dengue. Available: http://www.cdc.gov/dengue/symptoms/.
9. Crump JA, Morrissey AB, Nicholson WL, Massung RF, Stoddard RA, et al. (2013) Etiology of Severe Non-malaria Febrile Illness in Northern Tanzania: A Prospective Cohort Study. PLoS Negl Trop Dis 7: e2324. doi: 10.1371/journal.pntd.0002324 PMID: 23875053
10. Hertz JT, Munishi OM, Ooi EE, Howe S, Lim WY et al. (2012) Chikungunya and Dengue Fever among Hospitalized Febrile Patients in Northern Tanzania. Am J Trop Med Hyg 86: 171–177. doi: 10.4269/ajtmh.2012.11-0393 PMID: 22232469
11. Mayxay M, et al. (2013). Causes of non-malarial fever in Laos: a prospective study. Lancet Global Health 1(3): e46–e54. doi: 10.1016/S2214-109X(13)70008-1 PMID: 24748368
12. Powers AM and Logue CH. (2007) Changing patterns of chikungunya virus: re-emergence of a zoonotic arbovirus. J Gen Virol 88: 2363–2377. doi: 10.1099/vir.0.82858-0 PMID: 17698645
13. LaBeaud AD, Bashir F, and King CH. (2011) Measuring the burden of arboviral diseases: the spectrum of morbidity and mortality from four prevalent infections. Population Health Metrics 9: 1–11. doi: 10.1186/1478-7954-9-1 PMID: 21219615
14. Chanas AC, Hubalek Z, Johnson BK. & Simpson DI. (1979). A comparative study of o'nyong nyong virus with Chikungunya virus and plaque variants. Archives of Virology 59: 231–238. doi: 10.1007/BF01317418 PMID: 88212
15. Porterfield JS (1961). Cross-neutralization studies with group A arthropod-borne viruses. Bulletin of the World Health Organization 24: 735–741. PMID: 13737288
16. Karabatsos N (1985). International Catalogue of Arthropod-borne Viruses, 3rd edn, pp. 327–328, 767–768. San Antonio, TX: American Society of Tropical Medicine and Hygiene.

17. Sutherland LJ, Cash AA, Huang YJ, Sang RC, Malhotra I et al. (2011) Serologic evidence of arboviral infections among humans in Kenya. Am J Trop Med Hyg 85: 158–161. doi: 10.4269/ajtmh.2011.10-0203 PMID: 21734142

18. Bustinduy AL, Thomas CL, Fiutem JJ, Parraga IM, Mungai PL et al. (2011) Measuring Fitness of Kenyan Children with Polyparasitic Infections Using the 20-Meter Shuttle Run Test as a Morbidity Metric. PLoS Negl Trop Dis 5: e1213. doi: 10.1371/journal.pntd.0001213 PMID: 21750742

19. Bisanzio D, Mutuku F, Mungai L. Muchiri EM, King CH et al. (2014). Burden of Vector-Borne and Soil-Transmitted Polyparasitism in Rural Communities of Coast Province, Kenya. PLoS NTDs in press. PMID: 25057825

20. Pyke AT, Phillips DA, Chuan TF and Smith GA. (2004) Sucrose density gradient centrifugation and cross-flow filtration methods for the production of arbovirus antigens inactivated by binary ethylenimine. BMC Microbiol. 4:3. doi: 10.1186/1471-2180-4-3 PMID: 14720306

21. Chanas AC, Hubalek Z, Johnson BK, Simpson DI. (1979) A comparative study of O'nyong nyong virus with Chikungunya virus and plaque variants. Arch Virol 59(3): 231–8. doi: 10.1007/BF01317418 PMID: 88212

22. Kading RC, Borland EM, Cranfield M, Powers AM. (2013) Prevalence of antibodies to alphaviruses and flaviviruses in free-ranging game animals and nonhuman primates in the greater Congo basin. Journal of Wildlife Diseases 49(3): 587–599. doi: 10.7589/2012-08-212 PMID: 23778608

23. Brault AC, Foy BD, Myles KM, Kelly CL, Higgs S et al. (2004) Infection patterns of O'nyong nyong virus in the malaria-transmitting mosquito, Anopheles gambiae. Insect Mol Biol 13: 625–635. doi: 10.1111/j.0962-1075.2004.00521.x PMID: 15606811
